# Human Umbilical Cord Mesenchymal Stem Cells Prevent Bacterial Biofilm Formation

**DOI:** 10.1155/2022/1530525

**Published:** 2022-03-03

**Authors:** Haoming Yang, Fang Xu, Xuaner Zheng, Shumei Yang, Zhuxiao Ren, Jie Yang

**Affiliations:** Guangdong Women and Children Hospital, Guangzhou, China

## Abstract

Biofilm formation is easily found in patients suffered from ventilator-associated pneumonia (VAP) in neonatal intensive care unit (NICU) and makes the VAP infections not only harder to be treated but easier to relapse. In order to find some novel ways to inhibit biofilm formation, this study describe a previously unrecognized role for the human umbilical cord mesenchymal stem cells (hUCMSCs). In addition to multiple differentiation, hUCMSCs have the ability to prevent the biofilms formation in vitro by secreting antibacterial peptides (LL-37 and hBD-2). This occurred while *P. aeruginosa* PA27853 and hUCMSCs were cocultured, and the filtrated medium, which was the supernatant containing antibacterial peptides (5.9 ng/ml of LL-37, 1.77 ng/ml of hBD-2), and inhibited the growth of the bacterial biofilm on the surface of tracheal tube (2.5#, for preterm infant). Using microarrays, we were able to demonstrate that the antibacterial peptides from hUCMSC affected biofilm formation by downregulating the gene-encoded polysaccharide biosynthesis protein. In addition, in order to find out the most suitable concentration of hUCMSCs, *P. aeruginosa* was cocultured with eight-level concentrations of hUCMSCs, and we found that the concentration of LL-37 was positively correlated with the concentration of hUCMSCs. Meanwhile, the concentration of LL-37 became stable while the hUCMSC concentration reaches higher than 5 × 10^6^ cells/ml. But the concentration of hBD-2 had no significant correlation with hUCMSCs. The collection of these stem cells is not only limited by ethics but also reduces host rejection. This makes it possible to use autologous hUCMSCs to treat neonatal VAP.

## 1. Introduction

VAP is one of the common nosocomial infections in the NICU, and the incidence rate was about 5.2 per 1000 invasive ventilated patients and results in mortality of 23.5% [1]. In clinical practice, VAP is hard to treat and often causes significant morbidity and mortality. The biofilm on the surface of tracheal tube formed by antibiotic-resistant pathogen caused VAP hard to eradicate [2, 3]. Researches [4–6] report that biofilms are microbial communities encased in extracellular polymeric substances. The main components of biofilm are polysaccharides, proteins, lipids, and nucleic acids. Among them, extracellular polysaccharide substances (EPS) are considered as the skeleton [7]. EPS plays an important role in the formation of biofilm. EPS help bacteria adhere to the solid surface firmly and act as a barrier to protect bacteria [8]. The VAP associated with biofilm is hard to treat and easy to relapse [9]. Thus, we need a novel method to interfere the biofilm formation.

Human host defense peptides, also known as antimicrobial peptides, are secreted in response to infection, or the constant threat of infection. Human antimicrobial peptides, including cathelicidin hCAP-18/LL-37 and human *β*-defensin 2 (hBD-2), are ubiquitous in nature as components of innate immune defense systems [10]. Subinhibitory concentration of LL-37 (1/4–1/128 of the MIC of 64 *μ*g/ml) was shown to affect biofilm formation by decreasing the attachment of *P. aeruginosa* cells, stimulating twitching motility, and influencing two major QS systems (Las and Rhl), leading to the downregulation of genes essential for biofilm development [11]. A recent research suggested that both m-RNA and protein expression of cathelicidin hCAP-18/LL-37 in mesenchymal stem cells increased after bacterial challenge, which make mesenchymal stem cells possess direct antimicrobial activity [12].

Our previous researches show that hUCMSCs also have the ability to synthesize and secrete the LL-37 and HBD-2 in response to the *P. aeruginosa* [13]. However, researches about using autologous hUCMSCs to inhibit biofilm formation has not been found. Therefore, we designed experiments to find out the optimal concentration of antimicrobial peptides secreted by hUCMSCs and to test the hypothesis that hUCMSCs, by secreting antimicrobial peptides, possessed the ability to interfere the biofilm formation on the tracheal tube. Finally, the differences of *P. aeruginosa* gene expression were detected by microarrays to reveal the possible mechanism of hUCMSCs inhibiting biofilm.

## 2. Materials and Methods

### 2.1. Bacteria, hUCMSCs, Media, and Medical Catheters

Standard *P. aeruginosa* (ATCC27853) strains were offered by the Clinical Laboratory Centre of China's Ministry of Health. The experimental strains were kept at selective agar-plate at -4°C and grown at 37°C, 5% CO_2_ on blood agar plate. Before each experiment, the bacterial cells were resuspended in phosphate-buffered saline (PBS) and then adjusted to the concentration of 5 × 10^8^ CFU/ml through measuring optical density (OD at *λ* = 600 nm) of the suspension (according to the equation: OD_600_ = 0.5 corresponds to 5 × 10^8^ CFU/ml). For the cultivation of biofilms, *P. aeruginosa* PA27853 was grown at 37°C, 5% CO_2_ in complex Luria-Bertani (LB) broth [14]. hUCMSCs were purchased from American Type Culture Collection (ATCC; Manassas, VA). The cells met all the criteria for the classification as MSCs as defined by the International Society of Cellular Therapy. hUCMSCs were cultured at 37°C, 5% CO_2_ in DMEM/F12 medium (Gibeco, USA), comprising with 10% fetal bovine serum (FBS) on 25 cm^2^ cell culture flasks. Sterile tracheal tube (inside diameter 2.5 cm) was cut by sterile scissors into sections with length of 1 cm (according to the scale on the tracheal tube), 5 sections were used for experimental group, 5 sections were used for control group, and 2 for quality control.

### 2.2. Titration of hUCMSC Concentration

Seven target concentrations of hUCMSCs, diluted from the 5 × 10^8^ CFU/ml suspension, were included in the experiments (5 × 10^2^ CFU/ml, 5 × 10^3^ CFU/ml, 5 × 10^4^ CFU/ml, 5 × 10^5^ CFU/ml, 5 × 10^6^ CFU/ml, 5 × 10^7^ CFU/ml, 5 × 10^8^ CFU/ml). In coculture experiments, both bacteria cells and hUCMSCs were inoculated in the DMEM/F12 medium in polypropylene 6-well microtiter plates. After 6 hours of incubation at 37°C, the coculture supernatant was collected and filtered twice by 0.22 *μ*m filters. The concentration of LL-37 and HBD-2 in supernatant was determined by the ELISA kit from the Phoenix Pharmaceuticals and Hycult Biotechnology, respectively. All groups were carried out at least in three technical repeats.

### 2.3. Preparation of Supernatant after Coculture

The bacterial cells and hUCMSCs were cocultured at 50 ml cell culture flasks. One flask as experience group was inoculated with 30 ml of DMEM/F12 medium containing approximately 3 × 10^4^ cells/ml of *P. aeruginosa* PA27853 and 1 × 10^6^ cells/ml of hUCMSCs (according to the result of concentration titration). Another flasks, as the control group, were inoculated with 30 ml of DMEM/F12 medium containing 3 × 10^4^ cells/ml of *P. aeruginosa* and 1 × 10^6^cells/ml of normal human lung fibroblast (NHLF). After 6 hours of coculture (37°C, 5% CO_2_), DMEM/F12 medium from those two flasks was filtered twice by 0.22 *μ*m filters. The supernatant from experimental flask was marked as experience group, and the other was marked as control group.

### 2.4. Biofilm Experiment

*P. aeruginosa* biofilm was grown at 37°C, 5% CO_2_ in two polypropylene 6-well microtiter plates [14], in which 5 holes for experimental group, 5 holes for control group, and 2 holes for quality control. Each hole contains one sterile tracheal tube of 1 cm and 10 *μ*l of complex LB broth containing *P. aeruginosa* of 0.5 McF. The broth was replaced each 24 hours. After 4 days of incubation, the in vitro model of biofilm on tracheal tube was preliminarily established. Within next 3 days, those tracheal tubes (in vitro model) were incubated with filtrated medium as described above, and the filtrated medium was replaced each 24 hours.

After 7 days of incubation, the tracheal tube were rinsed three times with phosphate buffer solution (PBS), and the biofilm was fixed on the surface with formaldehyde. After crystal violet staining, the absorbance was measured in polypropylene 96-well microtiter plates at 595 nm [15].

### 2.5. Motility Assays

Swimming motility experiment was performed on semisolid plates containing 0.3% (wt/vol) agar. All bacterial motility evaluations have five technical repeats. After poured from the sterilized agar, the semisolid plates were dried in the biological safety cabinet at room temperature for 1 or 2 hours.

### 2.6. Statistical Method

SPSS (Ver.13) was used in this study as the statistical software. Product-moment correlation coefficient as the statistical method to analyze the correlation between the concentration of hUCMSCs and the level of antibacterial peptides, taking *P* < 0.05 for the difference was statistically significant.

### 2.7. DNA Microarray Experiment

The difference of gene expression were determined by microarray chip (Agilent, China). Microarray experiments were performed on those five independent cultures. The *P. aeruginosa* cells were harvested from the surface of tracheal tube. Only genes that exhibited a change of fivefold or more with a *P* value of ≤0.05 were considered in this study.

## 3. Results

### 3.1. Relationship between Antibacterial Peptide Levels and hUCMSC concentration

**Figure 1:**
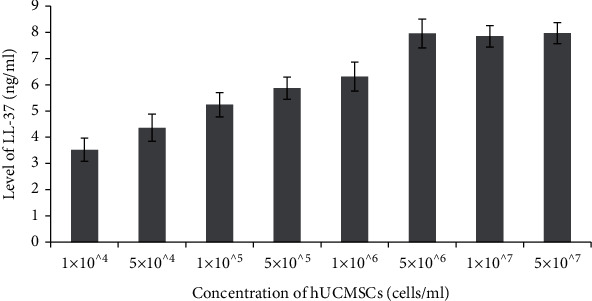
Level of LL-37 secreted by different concentration of hUCMSCs.

In this study, bacteria were cocultured with eight levels of hUCMSCs. The level of LL-37 increased significantly with increasing the concentration of hUCMSCs (Figure 1). The incremental effect did not appear while the hUCMSC concentration reach higher than 5 × 10^6^ cells/ml, and the highest level of LL-37 came from the 5 × 10^7^ hUCMSCs cells/ml. Since the concentration of LL-37 becomes stable while the hUCMSC concentration reach 5 × 10^6^ cells/ml, we excluded group 7 (1 × 10^7^ cells/ml) and group 8 (5 × 10^7^ cells/ml) and studied the correlation between the concentration of hUCMSCs and the level of LL-37. It indicated that the level of LL-37 in supernatant positively correlated with the concentration of hUCMSCs (Table 1).

**Figure 2:**
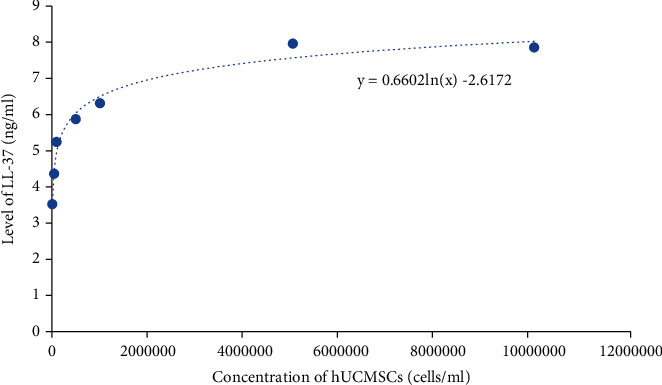
The log-linear regression line of LL-37 level versus concentration of hUCMSCs.

In addition, we excluded group 8 (5 × 10^7^ cells/ml) and tried to build the regression model of LL-37 level versus concentration of hUCMSCs.  Figure 2 suggested that the level of LL-37 in supernatant may increase with the concentration of hUCMSCs.

**Figure 3:**
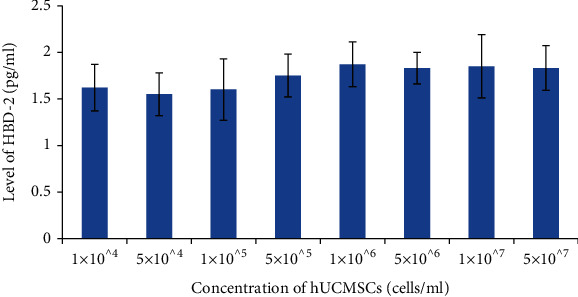
Level of HBD-2 secreted by different concentration of hUCMSCs.

The concentration of hBD-2 showed very low level similarly, but did not increased significantly with increasing the concentration of hUCMSCs (Figure 3). The highest level came from the group of 1 × 10^6^ hUCMSCs cells/ml. The statistical results indicated that the level of HBD-2 was not associated with the concentration of hUCMSCs (Table 2).

### 3.2. Concentrations of LL-37 and hBD-2 in the Coculture Medium

To test whether the inhibitory effect on biofilm formation was related to LL-37 and HBD-2 secreted by hUCMSCs in the filtrated medium, the concentrations of LL-37 and HBD-2 were measured after the coculture experiment using the ELISA kit. After stimulated by *P. aeruginosa*, hUCMSCs secrete LL-37 and HBD-2 in the DMEM/F12 medium. The difference was statistically significant different (Table 3).

### 3.3. Tracheal Tube Biofilm Analyses

**Figure 4:**
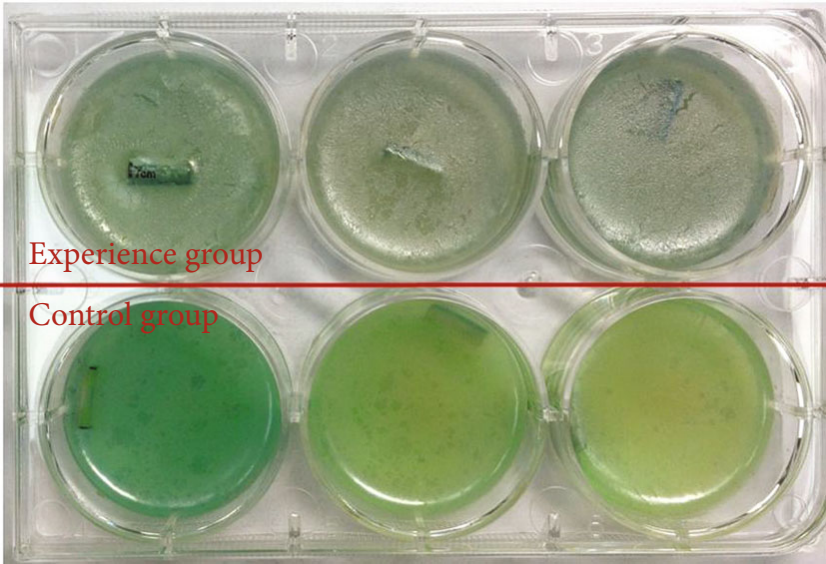
In vitro biofilm model established by tracheal tube. As the ability to produce green pigment, observing the color of culture medium would roughly judge the *P. aeruginosa* concentration. The concentration of pigment in the culture medium of the experimental group was significantly lower than that of the control group.

Since the artificial implants are usually performed as the solid surface in the VAP, tracheal tube was chosen as the solid surface for the bacterial attachment and the biofilms formation. After 3 days of incubation in presence of the supernatant, biofilm attached on the surface of tracheal tubes was stained (Figure 4) and measured the absorbance at 595 nm. Obviously, the biofilm cultured in the presence of LL-37 (5.90 ng/ml) and HBD-2 (1.77 ng/ml) was 56% less than the biofilm formed in the untreated control group (Table 4). The difference between two groups demonstrated statistically significant (Table 5).

### 3.4. Microarray Analysis

In order to investigate the mechanism of biofilm inhibition, microarray analysis was performed to examine gene expression profile of *P. aeruginosa*. The analysis demonstrated that greater than twofold change in expression levels of 106 genes downregulated (selected examples are shown in Table 6. All regulated genes are shown in the annex). The inhibiting effect of LL-37 at the concentrations of 16 g/ml (one-quarter MIC concentrations) has already been illuminated [11], and the peptides could influence gene expression of type IV pili and the quorum sensing systems in *P. aeruginosa*. However, the filtrated medium contains LL-37 of 5.9 *μ*g/ml and 1.77 ng/ml and seems to downregulate the gene expression of polysaccharide biosynthesis protein but not to influence type IV pili and the quorum sensing systems.

### 3.5. The Motility of Bacteria Was Not under the Influence

Low concentration of LL-37 (even at 4 *μ*g/ml) could stimulate bacterial surface motility. In the same way, we observed the motility of bacteria in presence of those two groups filtrated medium. Nevertheless, there was no difference about the motility between two groups (Table 7). The antibacterial peptides secreted by hUCMSCs may not stimulate motility of *P. aeruginosa.*

## 4. Discussion

Previous research [16] showed that the growth of colonies was inhibited in the cocultured in vitro model of bone marrow mesenchymal stem cells with Gram-negative bacteria. The possible mechanism is that the stem cells secrete more antimicrobial peptides after being stimulated by bacteria, thus presenting antibiosis effect. The major antibacterial peptides secreted by human epithelial tissues are LL-37 and HBD-2 [17], and their broad-spectrum antimicrobial effects on various bacteria have be confirmed [18, 19]. The hUCMSCs used in this study also secreted LL-37 and HBD-2 after stimulation, suggesting a possible bacteriostatic effect. The LL-37 in the supernatant of the experimental group can be found by protein electrophoresis [13]. Compared with NHLF, hUCMSCs can secrete more antimicrobial peptides into the filtrated medium. It is suggested that the ability of hUCMSCs to secrete antimicrobial peptides may be used in clinical practice.

Beside the bacteriostatic effect, the antibiofilm effect of antimicrobial peptides is also concerned. It has been proved that LL-37 can effectively inhibit the growth of biofilm, but the meaningful concentration (0.5 *μ*g/ml) [12] is significantly higher than that in these studies. Although the concentration of LL-37 measured in these experiments is only 5.9 ng/ml, which is significantly lower than that in previous study, similar inhibition effect can still be obtained. In the filtrated medium, the secretion of hBD-2 can also be detected. So, we may believe that LL-37 and hBD-2 can synergize to produce antibiofilm effect. More researches are needed to explore the mechanism that hBD-2 makes LL-37 produce antibiofilm effect at low concentration.

The formation of biofilm is affected by many factors. In this study, it was found that LL-37 (5.9 ng/ml) combined with hBD-2 mainly affected the expression of polysaccharide biosynthesis protein gene, thus reducing the formation of biofilm. But it did not significantly affect the bacterial attachment, mobility, and quorum sensing systems. Polysaccharide biosynthesis protein is one of the most common polysaccharides in bacteria. It has been proved that Polysaccharides in biofilm play as the role of skeleton and are important to biofilm [7]. If the ability of bacteria to secrete and synthesize polysaccharides is inhibited, the formation of biofilm is also affected. In addition, if the polysaccharide in biofilm can be reduced, the barrier effect on antibacterial drugs may be reduced.

In microarray analysis, it is found that the expression of fimbriae, flagella, and quorum sensing systems was not significantly affected. However, there are still numerous unknown genes that have been affected and downregulated (not listed in Table 5). The biological effects of these genes are not fully understood. We are curious about whether these genes are related to biofilm formation, so it is worth exploring the role of these genes.

As a novel antibiofilm pathway, the application of the hUCMSCs may have same obvious advantages especially in NICU. The Centers for Disease Control and Prevention (CDC; Atlanta, Ga., USA) defines VAP as “a nosocomial infection diagnosed in patients undergoing mechanical ventilation for at least 48 h” [20]. The formation of biofilm on the tracheal tube were easily to be found in preterm neonates suffering from VAP. In this study, biofilm models were established, in which biofilm was allowed to form on sterile surfaces for 96 hours (after 4 days of mechanical ventilation, the incidence of VAP increased significantly [21]). And the stem cell passage can be avoided in only 3 days of incubation within filtrated medium. Antimicrobial peptide secreted from hUCMSCs can effectively reduce the generation of biofilm on the surface of tracheal tube and indirectly reduce the occurrence of VAP probably. Expensive biomaterials are not needed in the extraction process, which highlights the advantages of economic benefits. In addition, only using the supernatant as an effective factor can avoid the possibility of exogenous tumor implantation during the direct use of stem cells for antimicrobial treatment.

On the other hand, it is already known that bone marrow mesenchymal stem cells have the ability to secrete antimicrobial peptides, but such stem cells were derived from foreign tissue, and the isolation process was subject to strict medical ethical restrictions, which might severely limit the clinical application. hUCMSCs are isolated from the umbilical cord, which would become the medical waste after the delivery. There will not be strict medical ethical restrictions in the application of hUCMSCs. In addition, the clinical use of allogeneic stem cells may cause severe rejection to the host, which will not only ineffective treatment but also aggravate the original condition. Obviously, hUCMSCs isolated from the host will be the effective way to solve the above problems.

Before applying hUCMSCs to prevent bacterial biofilm formation in vivo, we considered whether the concentration of hUCMSCs would affect the level of antibacterial peptide in supernatant. Since the relationship between the concentration of hUCMSCs and LL-37 has not been explored, this study set up experiments to explore the above issues. It was found that there was a good concentration correlation between LL-37 and hUCMSCs. It is obvious that high concentration of LL-37 can be obtained from high concentration of hUCMSCs (not higher than 5 × 10^6^ cells/ml), and the established concentration relationship model might serve as the data basis for future research. We also noted that the concentration correlation between hBD-2 and hUCMSCs is not significant. The possible reason is that hUCMSCs could secrete the highest concentration of hBD-2 at a lower concentration.

It is worth noting that higher concentration of hUCMSCs would be used to obtain higher concentration of LL-37; therefore, it may increase the cost of application. As the experimental group needed a large amount of supernatant (more hUCMSCs were needed to coculture), the concentration of 1 × 10^6^ hUCMSCs/ml was chosen in the coculture experiment under the condition of not affecting the experimental results. At the same time, we considered that hUCMSCs with higher concentration than 1 × 10^6^ cells/ml should also show the ability to inhibit the biofilm formation.

Previous research results [13] have showed that the filtrated medium could influence the formation of PA resistance. If combined with appropriate anti-infective course, application in vivo can reduce the dosage and use time of antibiotics and reduce the dependence on third-line antibiotics. However, it should be noted that the conditions of application in vivo is far more complex than in vitro. The distribution and metabolism of antimicrobial peptides in vivo will likely to affect the antibacterial effect. In addition, whether there are other potential biological effects of this filtrated medium, its mechanism have not been thoroughly studied. Therefore, researches using animal model in vivo still may help to fully understand the role of implant-related infection that the peptide can prevent.

## Figures and Tables

**Table 1:** The correlation between the concentration of hUCMSCs and the level of LL-37.

Groups^∗^	1	2	3	4	5	6
hUCMSCS (cells/ml)	1 × 10^4^	5 × 10^4^	1 × 10^5^	5 × 10^5^	1 × 10^6^	5 × 10^6^
LL-37 (ng/ml)	3.52	4.36	5.24	5.87	6.31	7.96
*r* = 0.853, *P* = 0.031						

^∗^Groups 7 and 8 were excluded.

**Table 2:** The correlation between the concentration of hUCMSCs and the level of LL-37.

Groups	1	2	3	4	5	6	7	8
hUCMSCS (cells/ml)	1 × 10^4^	5 × 10^4^	1 × 10^5^	5 × 10^5^	1 × 10^6^	5 × 10^6^	1 × 10^7^	5 × 10^7^
HBD-2 (pg/ml)	1.62	1.55	1.6	1.75	1.87	1.83	1.85	1.83
*r* = 0.409, *P* = 0.314								

**Table 3:** Concentrations of LL-37 and hBD-2 ($\bar{x}\pm s$).

Group	Cathelicidin/LL-37 (ng/ml)	*P* value^∗^	HBD-2 (ng/ml)	*P* value^∗^
Experimental group (hUCMSCs + PA27853)	5.90 ± 0.51	<0.001	1.77 ± 0.25	<0.001
Control group (NHLF + PA27853)	1.98 ± 0.34		0.72 ± 0.25	

^∗^Statistically significant level *P* ≤ 0.05.

**Table 4:** Absorbance of biofilm experiment.

Group/hole^#^	Absorbance	Mean ($\bar{x}\pm s$)
Experimental group (hUCMSCs + PA27853)		
1	0.152	0.214 ± 0.054
2	0.170	
3	0.210	
4	0.266	
5	0.270	
Control group (NHLF + PA27853)		
1	0.517	0.482 ± 0.043
2	0.418	
3	0.525	
4	0.486	
5	0.463	

^∗^33% glacial acetic acid was set as the reagent blank group. The situation that absorbance value are ≥ twice as reagent blank group is considered as biofilms formation [3].

**Table 5:** Absorbance statistic by Student's *t*-test.

Group	Cases (*n*)	Mean ($\bar{x}\pm s$)	*P* value
Experimental group	5	0.214 ± 0.054	0.000^∗^
Control group	5	0.482 ± 0.043	

^∗^Statistically significant level *P* = 0.05.

**Table 6:** Selected *P. aeruginosa* genes that were dysregulated.

Gene name	Fold increase	Protein
CP000744.1_cds_PSPA7_0598_597	-9.61	Allophanate hydrolase
CP000744.1_cds_PSPA7_2187_2163	-8.16	Probable hydrolase
CP000438.1_cds_PA14_23390_1869	-20.02	Polysaccharide biosynthesis protein
CP000744.1_cds_PSPA7_2314_2288	-11.10	Enoyl-CoA hydratase
CP000744.1_cds_PSPA7_5599_5524	-8.84	Termination factor rho

**Table 7:** The motility of *P. aeruginosa* ($\bar{x}\pm s$).

Group	Cases (*n*)	Mean (mm)	*P* value
Experimental group	5	23.7 ± 0.95	0.879^∗^
Control group	5	23.8 ± 0.84	

## Data Availability

Data will be available from the corresponding author if required.
